# Investigation of the Influence of Hot Forging Parameters on the Closing Conditions of Internal Metallurgical Defects in Zirconium Alloy Ingots

**DOI:** 10.3390/ma16041427

**Published:** 2023-02-08

**Authors:** Grzegorz Banaszek, Kirill Ozhmegov, Anna Kawalek, Sylwester Sawicki, Medet Magzhanov, Alexandr Arbuz

**Affiliations:** 1 Metal Forming Department, Częstochowa University of Technology, ul. J.H. Dąbrowskiego 69, 42-201 Częstochowa, Poland; 2 Mechanical Engineering Department, Abylkas Saginov Karaganda Technical University, 56 Nursultan Nazarbayev Ave., Karaganda 100027, Kazakhstan; 3 Core Facilities Department, Nazarbayev University, 53 Kabanbay Batyr Ave., Astana 010000, Kazakhstan

**Keywords:** hot forging mode, forging dies, zirconium alloy ingots, metallurgical discontinuities, plastometric studies, computer modeling

## Abstract

In this article, we present research results on the closing conditions of internal metallurgical discontinuities during the hot elongation operation of a Zr-1%Nb alloy ingot using physical and numerical modeling. Research on the influence of thermal and deformation parameters of elongation operations on the rheological behavior of a Zr-1% Nb alloy was conducted using the Gleeble 3800 metallurgical process simulator. Modeling of the influence of thermal–mechanical parameters of hot elongation operations in combinations of rhombic and flat anvils on the closure of metallurgical discontinuities was performed with the help of the FORGE^®^NxT 2.1 program. Based on the results of the research, recommendations were made regarding forging elongation technology and the geometry of working tools in order to ensure the closure of metallurgical discontinuities during hot elongation operations of Zr-1% Nb alloy ingots.

## 1. Introduction

Zirconium-based alloys are widely used for the production of structural parts operating under extreme conditions, including fuel rods for nuclear reactor cores [1,2,3]. Zirconium alloy parts are subject to strict requirements, primarily with respect to mechanical and corrosion properties, as well as to ensure the uniformity of their structure and a lack of internal metallurgical discontinuities [2]. The presence of internal metallurgical discontinuities in structural parts may lead to faulty operation (shortening working cycles of fuel rods); the most negative effect is the expansion of fuel rods with coolant outflow (LOCA failure) [4].

A Zr-1%Nb (M5) alloy is a basic alloy used for the production of fuel rod parts (shirts and final elements) for PWR reactors [5].

Manufacturing technology for the production of round Zr-1%Nb alloy parts includes the double vacuum–arc remelting of ingots, hot ingot forging, hot billet pressing with or without piercing, several cycles of cold pilger rolling for semi-finished products, and the intermediate and final heat treatment of finished products [2]. In the smelting process, the formation of a shrinkage cavity is inevitable due to the crystallization rate gradient over the ingots’ volume, as well as the formation of gas porosity [6,7]. After smelting, ingots undergo ultrasonic continuity testing, and such shrinkage cavity defects are detected and removed. However, based on our experience, at a distance of 1.2R, gas pores with axial dimensions of up to Ø10 mm and radial dimensions of up to Ø1.0 mm are not eliminated by this process.

We conducted a literature analysis [8,9,10,11,12,13,14,15,16] to explore methods of closing internal metallurgical discontinuities in the process of the hot forging of steel ingots. We previously conducted the numerical modeling of the welding of metallurgical discontinuities during the rod-rolling process in Mg alloy blanks using the FORGE program, and physical verification results were presented in [17]. The numerical results were confirmed by tests under laboratory conditions. However, no studies have been carried out to date on closing internal metallurgical discontinuities in the hot forging process in zirconium alloy ingots. Therefore, in the present study, the closing conditions for internal metallurgical discontinuities were investigated, and recommendations were developed for the mode of the hot forging of zirconium alloy ingots [18].

According to [2,3,19], the hot forging of zirconium alloy ingots is carried out according to the temperature range of the α + β and β zones. Under these conditions, the metal can be subjected to significant deformations without the formation of internal defects. The studied alloy crystal structure can change depending on the polymorphic transformation temperature. Up to temperatures of T = 862 °C, the crystal lattice is HCP. Above this temperature, it transforms to a BCC state. An important aspect is the metal surface oxidation that occurs during the heating and forging processes in the natural atmosphere, especially with respect to the hydrogen pickup process. Rapid heating is desirable, especially for temperatures above 800 °C, because under these conditions, the gas absorption rate rapidly increases. If possible, it is advisable to use protective coatings when metal is heated for forging and to ensure sufficient metal removal during the machining of forgings.

## 2. Purpose and Scope of the Work

In this paper, we present guidelines for the development of forging elongation technology during the forging of zirconium alloy ingots in order to ensure the welding of internal metallurgical discontinuities in ingots. Ingots were obtained through a process of double vacuum melting in an arc furnace. In order to achieve the purpose of the work, we proposed that forge elongation operations be carried out under hot conditions using flat and rhombic anvils.

In order to develop technological guidelines, numerical tests of the ingot forging process were carried out using three different anvil compositions, including two shaped anvils.

The elongation operation was modeled using the Forge^®^NxT 2.1. program based on MES, which allows for the tracking of changes in hydrostatic pressure distributions and deformation intensity in plastically processed metal during any stage of the elongation operation [20]. The distributions of hydrostatic pressure and the intensity of deformation on the cross-section of the deformed Zr alloy after each forging pass were determined.

During model tests of forging elongation operations, the results of plastometric tests of a given Zr alloy were used, based on which, graphs of the dependence of the stress on the deformation of the actual Zr alloy were developed, and according to which, the coefficients of the flow stress function were selected.

Based on the analysis of the results of numerical research, guidelines for forging elongation technology were developed in order to weld internal metallurgical discontinuities with flat and rhombic anvils.

The test results reported herein can contribute to the improvement of the structural and mechanical properties of Zr alloy rods, supporting the appropriate selection of anvil shapes.

## 3. Methodology of Plastometric Research

The purpose of the plastometric tests was to determine the coefficients of the deformation resistance function, taking into account the influence of temperature and strain rate. The determination of these coefficients is necessary for numerical calculations using the finite element method. We cannot to carry out relevant modeling without these data.

The material used for the research was an M5 alloy with the following chemical composition: Zr–1.1%Nb–0.05%Fe—0.6%O.

In order to determine the flow stress σ_p_ of the M5 alloy in the conditions of multi-transition hot forging, an experimental diagram was developed (Table 1), which takes into account the temperature T change and the deformation value ε. According to the diagram shown, the maximum value of the actual deformation is 0.9, and the temperature value varies from 770 °C to 950 °C. The range of forging temperature is described in [2,19,21]. According to the Zr-Nb phase equilibrium system, at a temperature of 870–950 °C, the metal structure is characterized by grains shaped as an elementary cell A2. In the range of forging temperatures of 750–870 °C, the structure of zirconium is in the area of α + β [2]. When the sample is deformed from the Zr–1% Nb alloy within a certain temperature range, the deformation limit decreases 2-fold [22]. The authors’ experience shows that forging this alloy at temperatures below 750 °C leads to the occurrence of cracks in forging.

The average strain rate during the hot forging of the zirconium alloy ingot on the hydraulic press is 0.5 s^−1^ [19]. However, in local areas of the deformed rod in the form of anvils, the strain rate is slightly higher. Therefore, for the practical use of the results of physical modeling, the range of deformation velocities used for the tests was from 0.5 s^−1^ to 5.0 s^−1^.

Mechanical properties were tested using the Gleeble 3800 plastometer with the Pocket Jaw module. The tests were performed by compressing cylindrical samples of M5 zirconium alloy with diameters of 10 mm and 12 mm lengths after recrystallization.

The main advantages of this method include the possibility of obtaining relatively large deformation values (up to ε = 1.2); in addition, it is the most favorable deformation state to describe the rheological properties of a material during plastic deformation [23].

In order to make practical use of the results of plastometric tests of the M5 zirconium alloy, an approximation of flow curves was carried out σ_p_−ε using the generalizing relationship—functions of Henzel A., Spittel T. [24]:

σ_p_ = A·e^m1·T^·T^m9^·έ^m2^·e^m4/ε^·(1 + έ)^m5·T^·e^m7·ε^·έ^m3^·έ^(m8·T)^ (1)

where σ_p_—flow stress, T—temperature of the deformed material, ε—actual deformation, έ—strain rate, A, m1–m9—function coefficients.

The approximation of the test results was carried out according to the method presented in [14] in the FORGE^®^NxT 2.1 ”Rheology Database” program at the Faculty of Production Engineering and Materials Technology of the Czestochowa University of Technology.

## 4. Methodology of Numerical Research

The paper analyzed the elongation operation of zirconium alloy rods for three anvil compositions, including rhombic anvil compositions and rhombic flat anvil compositions. The numerical modeling of the elongation operation was carried out in order to analyze the possibility of welding metallurgical discontinuities in zirconium alloy rods for individual rhombic and flat anvil compositions. Each of the anvil compositions was characterized by a different geometric surface of the deformation basin. The variable geometric surface of the deformation basin resulted in different directions and reversals of the pressure and friction forces in the deformed material in the deformation zone, which had an impact on the welding mechanism of the modeled metallurgical discontinuities inside the deformed rods. The constant parameters of the elongation operation were: forging start temperature, relative crush value, and application angle value, while for individual operations, the type of composition used in the incubators was changed.

A commercial computer program on the PC FORGE^®^NxT 2.1, a product of Transvalor Solution, was used to model the elongation operation in three anvil compositions in order to achieve the welding of metallurgical discontinuities during the deformation of the zirconium alloy rod. This program allows for the thermomechanical simulation of, among others, plastic processing. A detailed description of temperature, energy, strain, and deformation functions, as well as thermomechanical and friction laws used during the calculations, can be found in [25]. For thermal calculations, the Galerkin equation built into the program was used, while the reinforcement curves were approximated using the Henzel–Spitel Equation (1) [26], which is also built into this program. FORGE^®^NxT 2.1. is based on the finite element method (FEM). In the paper, to simulate the elongation operation, a thermo-viscoplastic model of the deformed body, which is based on the theory of large plastic deformations, was used. To generate the grid of finite elements, tetrahedral elements with a base of triangles were used. In the model input created in this way, the number of nodes equal to the range of 8900 ÷ 9500 was assumed for the simulation, while the number of tetrahedral elements was in the range of 40,700 ÷ 42,000. The value of the coefficient of friction between the anvil surface and the deformed rod was adopted in accordance with the Coulomb law, and it was μ = 0.3. It was assumed that the heat transfer coefficient between the anvils and the material was λ = 10,000 W/m^2^K, while the heat transfer coefficient between the metal and the environment was equal to λ = 10 W/m^2^K. The ambient temperature was assumed to be 25 °C, while the anvil temperature was assumed to be 350 °C. The initial temperature of the model rod was 950 °C. In all the forging transitions, 35% relative compaction was adopted. The feed rate of the upper anvil was v = 8 mm/s, while the lower anvil was assumed to be stationary.

The diffusion model is not included in FORGE^®^NxT 2.1. The inference of discontinuous welding is based on the values of hydrostatic pressure and the temperature of the bar being elongated. Therefore, in the elongation operation, the goal is to achieve the maximum possible values of hydrostatic pressure within the existing defects, as well as to maintain a high temperature close to the temperature at the beginning of forging. During the welding process of the discontinuity, on the right and left sides of the discontinuity, there are medium stresses with large values; these are generally negative medium stresses (there is high hydrostatic pressure). On the other hand, at the top and bottom of the welded discontinuity, unfavorable positive medium stresses with very high values are formed (there is no hydrostatic pressure) [27].

Zirconium alloy rods with diameters of 100 mm and lengths of l = 50 mm were deformed in each of the three anvil compositions in four forging transitions.

**Figure 1:**
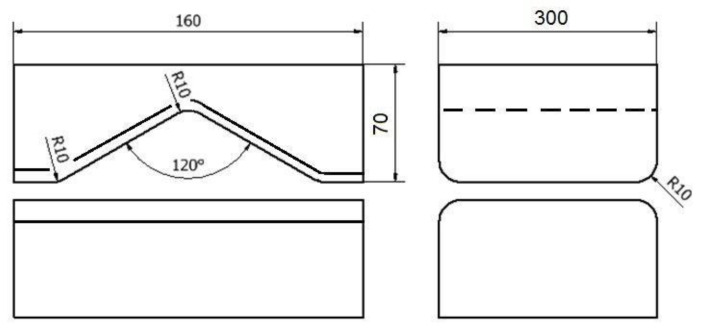
Shape and dimensions of rhombic flat anvils used to deform the zirconium alloy.

**Figure 2:**
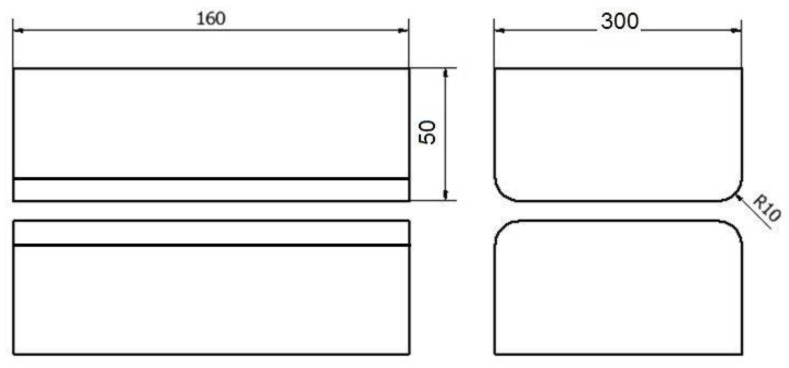
Shape and dimensions of flat anvils used to deform the zirconium alloy.

**Figure 3:**
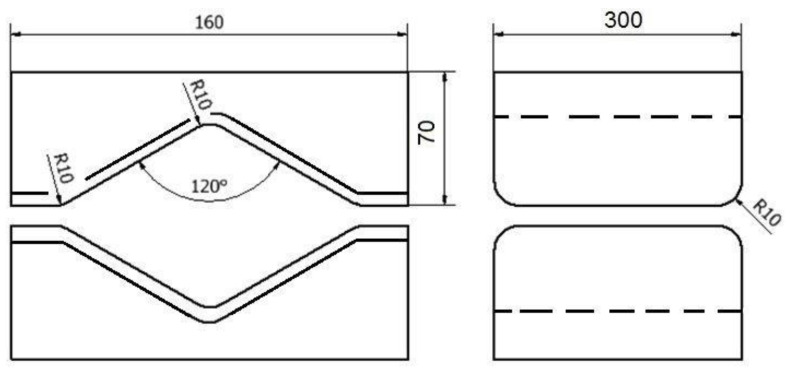
Shape and dimensions of rhombic anvils used to deform the zirconium alloy.

The shape and dimensions of the anvils used in the work are presented in Figure 1, Figure 2 and Figure 3. The rod was elongated in four forging transitions. For example, the paper describes the diagram of conducting elongation operations in rhombic flat anvils (Figure 1). In the first forging transition, rhombic flat anvils were used, in which a rod previously heated to 950 °C with a relative 35% crumple was deformed, and then, the rod was rotated 90° clockwise. In the second forging transition, after rotating the rod with the same anvils, a 35% crumple was made again, and it was rotated 90 clockwise. Then, before the third forging transition, the rod was heated to 950 °C, because after the second forging transition in local areas, the temperature of the rod fell to 750 °C, and in accordance with the forging practice of zirconium alloys, it is impossible to carry out further stages of the elongation operation below the described temperature. Then, the third and fourth forge transitions were already implemented in flat anvils (Figure 2) with a 35% crumple. After the third forging transition, the rod was rotated 90° clockwise.

For the assembly of rhombic anvils, they were treated in a manner analogous to the forging scheme described above (Figure 3). Shape anvils were used in the first two forging transitions, and in the other two flat anvils, the exception was the pattern of forging in flat anvils, when only flat anvils were used in all four stages. Each time after the first two forging transitions, a forged rod was heated for all three of the forging schemes analyzed in the paper.

**Figure 4:**
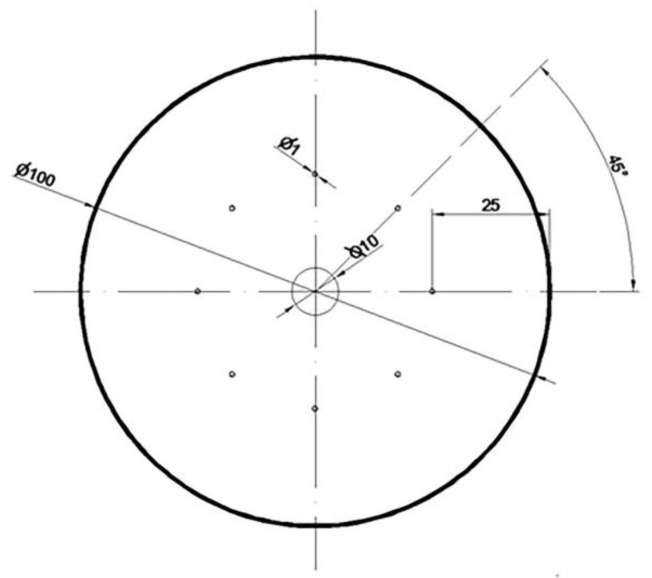
Shape and dimensions of the modeled metallurgical discontinuities on the front surface of the model zirconium alloy rod.

The shape, dimensions, and arrangement of the modeled metallurgical discontinuities on the front surface of the model zirconium alloy rod are presented in Figure 4.

The model input for the elongation operation was a cylinder with a diameter of 100 mm and a length of 50 mm, in which nine metallurgical discontinuities with an input length of 50 mm were modeled; the first axial one had a diameter of 10 mm, and the eight remaining discontinuities with diameters of 1 mm were distributed around the perimeter of the model input at a distance of 25 mm from its axis. The input modeled in this way was a reflection of the ingot obtained after the process of double vacuum melting in an arc furnace. On the other hand, artificially modeled holes were used to simulate internal casting discontinuities such as middle porosity and casting voids. A detailed description of this type of defect can be found in the literature [28,29,30,31].

The model input and anvils were designed in the commercial AutoCad Mechanical 2009^®^ program, which is a product of Autodesk. Metallurgical discontinuities were drawn as rollers inside the model input. Using the “difference” tool in AutoCad, metallurgical discontinuities were understood by the program as holes in the model input. The model input drawing with artificially simulated metallurgical discontinuities was exported to FORGE^®^NxT 2.1 by the file format “*.stl”. The same actions were carried out for all anvil compositions. In the FORGE^®^NxT 2.1 program, using the “stlmeshing” and “volumemeshing” tools, a 2D triangular mesh was applied to the input and artificially modeled discontinuities, followed by a 3D quadrilateral mesh. In addition, in order to properly simulate the closure of discontinuities and their correct formation, the built-in “FoldsDetection”, “SelfContact”, and “Damage” tools were used. Thanks to the use of the presented tools, during the FEM simulation, in the areas of welded discontinuities, there were no computational errors or degeneration of the grid. When welding discontinuities, the nodal points of the quadrilateral elements in close contact connect with each other, and thus, the modeled metallurgical discontinuity is eliminated. Thanks to such an adopted modeling methodology, there were no errors in the form of the penetration of the nodes of tetrahedral elements around the closed discontinuities.

Based on the results of numerical simulations of the welding process of metallurgical discontinuities during the operation of elongating Zr alloy rods in flat and rhombic anvils, the volume values of the remaining unwelded discontinuities were determined. The procedure was as follows: after the simulation of the elongation operation was completed, a file with a deformed rod datum in the “*.stl” format was exported in FORGE^®^NxT 2.1 to RinoCeros 3.0^®^; then, the grid of tetrahedral elements was separated in this program, and the elements of the rod outline were removed. The next step was to merge the previously broken grid of tetrahedral elements (then, only the elements constituting the contour of discontinuity). In the last step, using the “physical parameters” tool in RinoCeros 3.0^®^, the total volume of discontinuities for individual elongation operations was obtained, which is shown in Figure 4.

After the elongation operations of the rods in three anvil assemblies, the hydrostatic pressure values at the nodal points of the tetrahedrons constituting the contour of unheated discontinuities were determined as the arithmetic mean of the occurring stresses. An analogy was made for the deformation intensity values. However, all the results presented in the paper are burdened with a certain error resulting from numerical errors and generalizations used in retrieving the data needed to analyze the results obtained.

The authors analyzed the distributions of strain intensity and hydrostatic pressure only after the first and second forging passes, since in the third forging pass, all of the metallurgical discontinuities were closed for all anvil geometry options.

## 5. Testing Results

**Figure 5:**
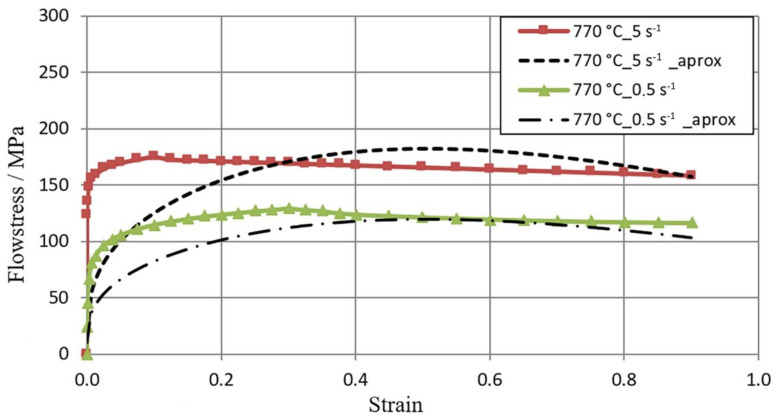
σ_p_−ε M5 alloy flow curves obtained for the temperature T = 770 °C in the range of έ strain rate from 0.5 to 5.0 s^−1^ using the Gleeble metallurgical process simulator 3800; (1) continuous lines—experimental data; (2) dashed lines—data after approximation.

**Figure 6:**
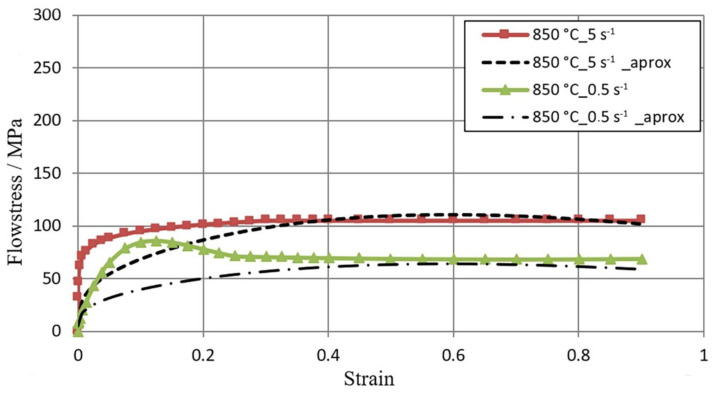
σ_p_−ε M5 alloy flow curves obtained for the temperature T = 850 °C in the range of **έ** strain rate from 0.5 to 5.0 s^−1^ using the Gleeble metallurgical process simulator 3800; (1) continuous lines—experimental data; (2) dashed lines—data after approximation.

**Figure 7:**
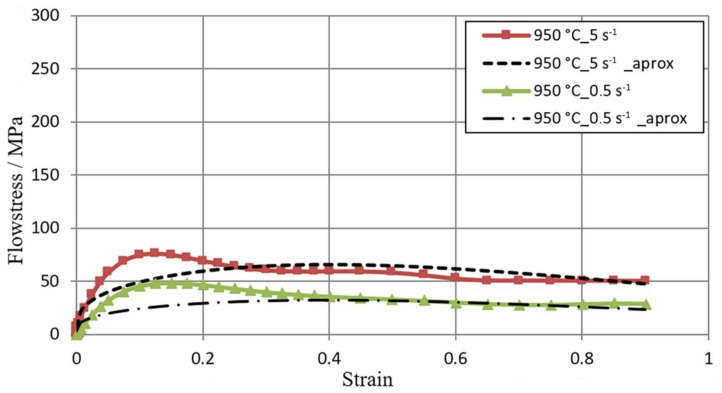
σ_p_−ε M5 alloy flow curves obtained for the temperature T = 950 °C in the range of **έ** strain rate from 0.5 to 5.0 s^−1^ using the Gleeble metallurgical process simulator 3800; (1) continuous lines—experimental data; (2) dashed lines—data after approximation.

Figure 5, Figure 6 and Figure 7 show the flow curves of the M5 alloy deformed at T = 770–950 °C for the strain rate in the range **έ** from 0.5 s^−1^ to 5.0 s^−1^, obtained using the Gleeble 3800 plastometer. Data in Figure 5, Figure 6 and Figure 7 show that with the increase in the temperature of samples T from 770 °C to 950 °C, the value of the flow stress σ_p_ decreased about 2.5 times. An increase in the strain rate from 0.5 s^−1^ to 5.0 s^−1^ led to an increase in the value of the flow stress σ_p_. At T = 770 °C, this increase was about 29%, while at T = 850 °C, this increase was about 36%, and at T = 950 °C, it was 43%. Thus, the effects of increasing strain rate on flow stress σp with increasing test temperature were accelerated. Such an effect could have a connection with the decrease in the plastic deformation thermal effect with regard to the test temperature acceleration [23].

Table 1 shows the s^−1^ over the entire tested temperature range; the flow curves σ_p_−ε reached the maximum stress value for a given deformation value, whereby as the temperature increased, the maximum flow stress value σ_p_ moved in the direction of smaller actual deformation values.

The data presented in Figure 6 show that the flow curves obtained at the strain rates **έ** = 0.5 s^−1^ and **έ** = 5.0 s^−1^ were of a different nature. The maximum value of flow stress σ_p_ at the strain rate equal to έ = 0.5 s^−1^ was obtained for the value of actual deformation equal to **ε** = 0.12, and with a further increase in deformation, a decrease in the value of flow stress σ_p_ was observed. For the strain rate **έ** = 5 s^−1^, the value of flow stress σ_p_ increased to the value of actual deformation ε = 0.3, after exceeding which, the plateau effect was observed on the curve.

This difference may have been due to the fact that as the strain rate increases, the process of dynamic softening is delayed. It is possible that dynamic recrystallization is inhibited and the softening of the metal proceeds in accordance with the mechanism of dynamic polygonization. In addition, at a temperature of T = 862 °C, a polymorphic transformation took place in the zirconium, when the crystalline network changed from hexagonal A3 to a regular spatially centered network A2 [2]. Therefore, the non-monotonicity of the flow curve in the tested deformation velocity range could have been caused by the beginning of the polymorphic transformation [23].

Figure 5, Figure 6 and Figure 7, in addition to experimental data, also present the results of the approximation of experimental tests of flow curves. It is assumed that the coefficients of the approximating function (1) are sufficiently well-selected if the average approximation error does not exceed 15% [32]. For the range of thermo-mechanical parameters analyzed in the paper, the average approximation error was about 10%. As a result of the approximation of experimental M5 alloy flow curves, Hensel–Spittel equation coefficients were determined for the following temperature and strain rate conditions of plastic processing: T = 770–950 °C; έ = 0.5–5.0 s ^−1^ (Table 2).

**Figure 8:**
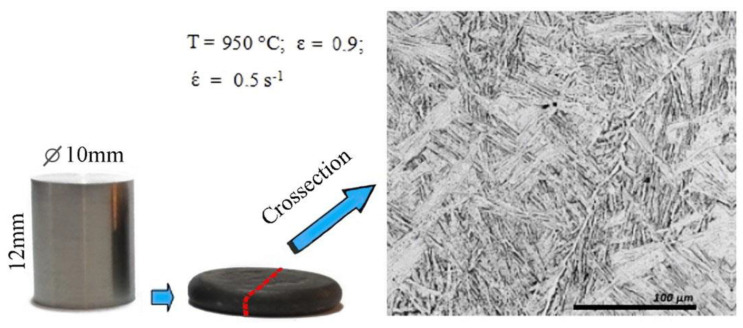
Sample for plastometric study Ø10 × 12 mm of M5 alloy and its structure after plastometric testing at T = 950 °C and έ = 0.5 s^−1^.

Figure 8 shows the sample before and after the plastometric test on the example of a sample tested at T = 950 °C and its structure. The sample was cut across, and a cross-section was prepared. Finishing, polishing, and etching were carried out using the electrolytic method at the LectroPol-5 (Struers) installation in the A3-branded electrolyte recommended by the manufacturer (Struers). As can be seen, under conditions of hot deformation, the sample lends itself to significant deformations (up to ε = 0.9) without destruction. The structure after cooling is a Widmanstatt type.

## 6. Analysis of Hydrostatic Pressure Distribution during Elongation Operations

**Figure 9:**
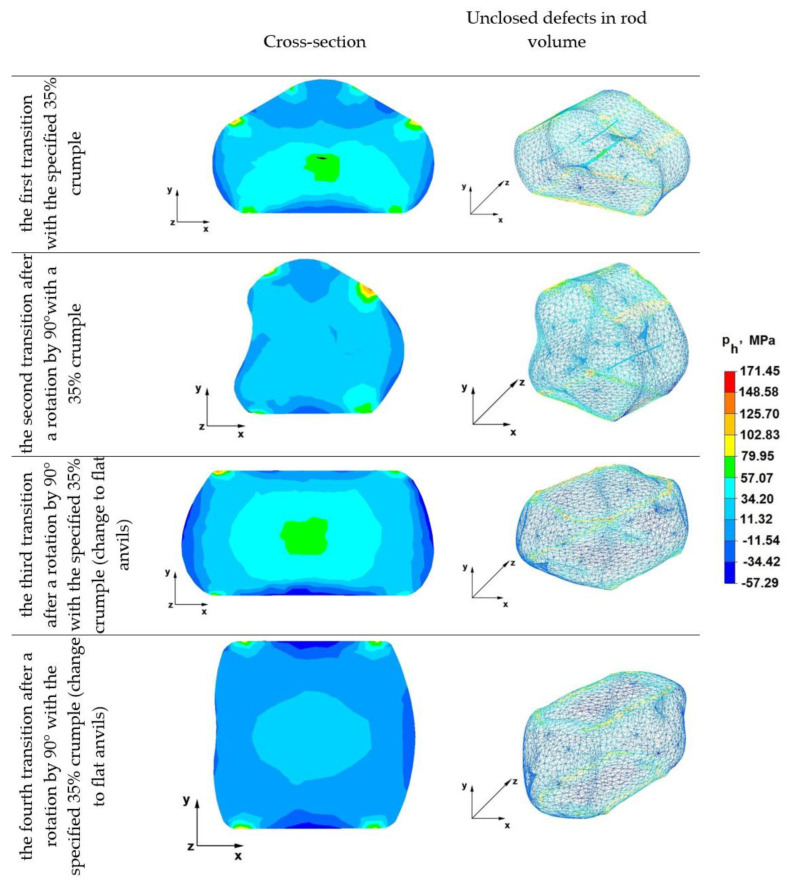
Hydrostatic pressure distribution in rhombic flat anvils.

The results of the research on the distribution of hydrostatic pressure during the operation of elongating the Zr alloy rod in various anvil compositions are presented in Figure 9.

### 6.1. Analysis of the Distribution of Hydrostatic Pressure Values during Elongation Operations in Rhombic Flat Anvils

Figure 9 shows the distribution of hydrostatic pressure values obtained during numerical simulations of the operation of lengthening the rod from the Zr alloy in rhombic flat anvils.

The data of the first transition in Figure 9 show that in the axial zone of the forged rod in the first forging transition, favorable hydrostatic pressure values of about 57 MPa were obtained, but nevertheless, the axial artificially modeled discontinuity was not completely welded. Hence, it follows that for the complete heating of discontinuities with a diameter of about 10 mm, the value of the hydrostatic pressure in this zone should be higher. There was also no complete welding of the three discontinuities located in the upper part of the forged rod in the upper rhombic anvil impact zone. The hydrostatic pressure in the areas containing these discontinuities reached values in the range of 11 to 34 MPa. On the other hand, all discontinuities located in the lower part of the forged bar, under the action of the lower flat anvil, where the pressure value was 34 MPa, were completely welded.

On the basis of the test results presented in the second transition, it can be stated that after rotating the forging by 90° in a clockwise direction and deforming again with a relative 35% crumple, the middle discontinuity with a diameter of 10 mm was completely welded, thanks to the appropriate direction of pressure and friction forces directed in the direction of the rod axis, which was achieved by using various working surfaces of the bottom flat anvil and the upper anvil. The value of hydrostatic pressure in the axial area of the forged rod, as well as in the areas adjacent to it, was even and amounted to 34 MPa. Only one of the eight breaks with a diameter of 1 mm on the right side of the forged rod was not welded, as can be seen in the image of the volume bar in the second transition. This discontinuity in the first forging transition was located under the apex of the upper rhombic anvil, which indicates that in both the first and second forging transitions, the values of compressive stresses and compressive deformations resulting from the interaction of the upper rhombic anvil were insufficient to close even small discontinuities with a diameter of 1 mm. It is also worth noting that in the first forging transition from the side of the impact of the rhombic anvil, not only the described discontinuity but also two others, located in its close area, were not welded.

The distribution of hydrostatic pressure after the deformation of the rod during the third forging transition with a relative 35% crumpling after turning the forging by 90°, preheated to 950 °C, showed that none of the simulated inhomogeneities were visible in the volume of the rod, which means that after heating the forged zirconium alloy rod and using cullet in flat anvils, all the irregularities were completely welded. As can be seen in the figure, the hydrostatic pressure values in the middle area of the forged rod in flat anvils were in the range of 34–57 MPa, and in the area adjacent to the middle, they were 11 MPa. Such a distribution of hydrostatic pressure values occurring on the cross-section of the forged bar is beneficial because it causes the welding of all metallurgical discontinuities.

By analyzing the data of the fourth transition, it can be stated that in the last fourth elongation operation, the distribution of hydrostatic pressure values on the cross-section of the forged bar was beneficial in terms of obtaining conditions for the welding of discontinuities. It is known that discontinuities with larger diameters are more difficult to seal than discontinuities with smaller diameters. In the elongation operations analyzed in the paper, higher hydrostatic pressure values were observed in the axial zones of the forgery, and in the zones adjacent to the axial zone of the lower value, and so in the axial zone, the pressure values ranged from 11 to 34 MPa, and in the zones adjacent to the external surfaces, they ranged from 7 to 11 MPa. The exceptions were zones with a small area of contact with anvils, where there was no hydrostatic pressure. The hydrostatic pressure value achieved in the outer zones of the elongated rod was irrelevant, as these zones constitute a technological loss for machining.

### 6.2. Analysis of the Distribution of Hydrostatic Pressure Values during Elongation Operations in Rhombic Flat Anvils

For a more accurate analysis of the differences in the distribution of hydrostatic pressure along the forging passes, the authors did not use the same legends for each version of the anvils’ geometry. For each anvil geometry variant, there is a different distribution of hydrostatic pressure, which depends on the direction and magnitude of the forging force and the friction force associated with different anvil surfaces. According to the authors, the use of one legend for all variants of the geometry of the strikers would lead to too much generalization of the hydrostatic pressure values and would hide the differences.

**Figure 10:**
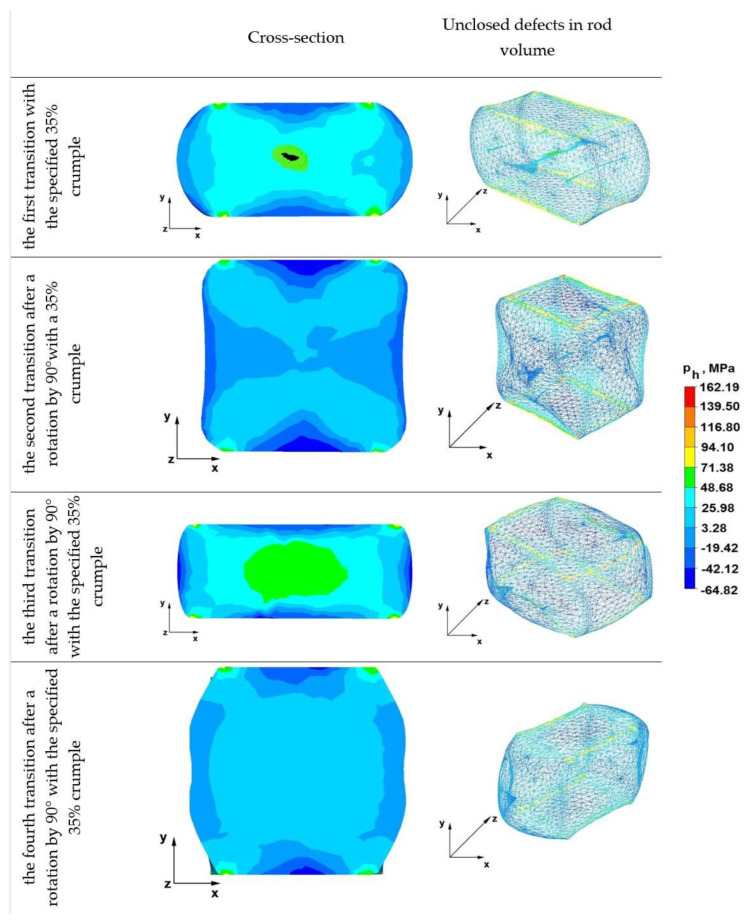
Hydrostatic pressure distribution in rod elongation operations in flat anvils.

Figure 10 shows distributions of hydrostatic pressure values obtained during numerical simulations of the Zr alloy rod elongation operations in flat anvils.

From the analysis of the data of the first transition shown in Figure 10, it follows that the axial discontinuity and the two external discontinuities located on the right and left sides of the forged rod were not welded. Where axial discontinuity occurred, the hydrostatic pressure value was 49 MPa, while where other discontinuities occurred, the pressure value was in the range of 15–26 MPa. The discontinuities in the right and left area of the forged rod did not become welded, because the metal floated freely along the x-axis without being limited by the side surfaces of the shaped anvils, as was the case with forging in anvils, e.g., rhombic flat anvils. On the other hand, all discontinuities in the areas of the lower and upper forged rods under the direct action of flat anvils were welded, because there was a favorable distribution of compressive stresses, directed along the y-axis in the direction of the rod axis.

From the data of the distribution of hydrostatic pressure values during the elongation operation in flat anvils, it appears that after the second forging transition, there was still no complete welding of discontinuities remaining after the first transition. It is worth noting that it is advantageous to use rhombic flat anvils, thanks to which, the discontinuity located in the axis of the deformed rod was completely welded after the second transition (second transition in Figure 9). Two discontinuities lying in the zone outside the axis of the rod, in the second forging transition, were not completely heated, because no hydrostatic pressure was found in these areas, and the average stress values were in the range of 19–42 MPa. Additionally, the axial discontinuity was not completely welded, because the hydrostatic pressure value was lower than during the forging in the first transition. In the second forging transition, the hydrostatic pressure value in this area fell from 49 MPa to 26 MPa and below. It is likely that if the high hydrostatic pressure from the first transition is maintained, the axial discontinuity would be completely welded, and with a significant reduction in the value of this pressure, this defect was welded in half.

Before the third forging process, the rod was heated to 950 °C and rotated 90°, after which, 35% relative creasing was carried out. In this case, in the axial zone of the deformed rod, the hydrostatic pressure value was 49 MPa, while in the zones adjacent to the axial area of the rod, it was about 26 MPa. In the third forging transition carried out in flat anvils, there were no discontinuities. This is a beneficial effect because the authors’ aim was to find the best possible parameters of the technological process of shaping the zirconium alloy by means of forging in order to completely seal the metallurgical discontinuities in the ingot after the casting process.

By analyzing the data in Figure 10, it can be stated that in the cross-section of the deformed rod after the fourth, last forging transition, an even character of the hydrostatic pressure distribution was obtained, the value of which was 26 MPa. The exception was only small areas near the contact zone of tools with the material and boundary zones on the right and left side of the rod, where the material moved freely along the x-axis. The pressure values were 3MPa or negative, which means that there was no hydrostatic pressure. However, this does not matter, as these zones are a technological defect for subsequent machining and surface treatment.

### 6.3. Analysis of the Distribution of Hydrostatic Pressure Values during the Elongation Operation in Rhombic Anvils

**Figure 11:**
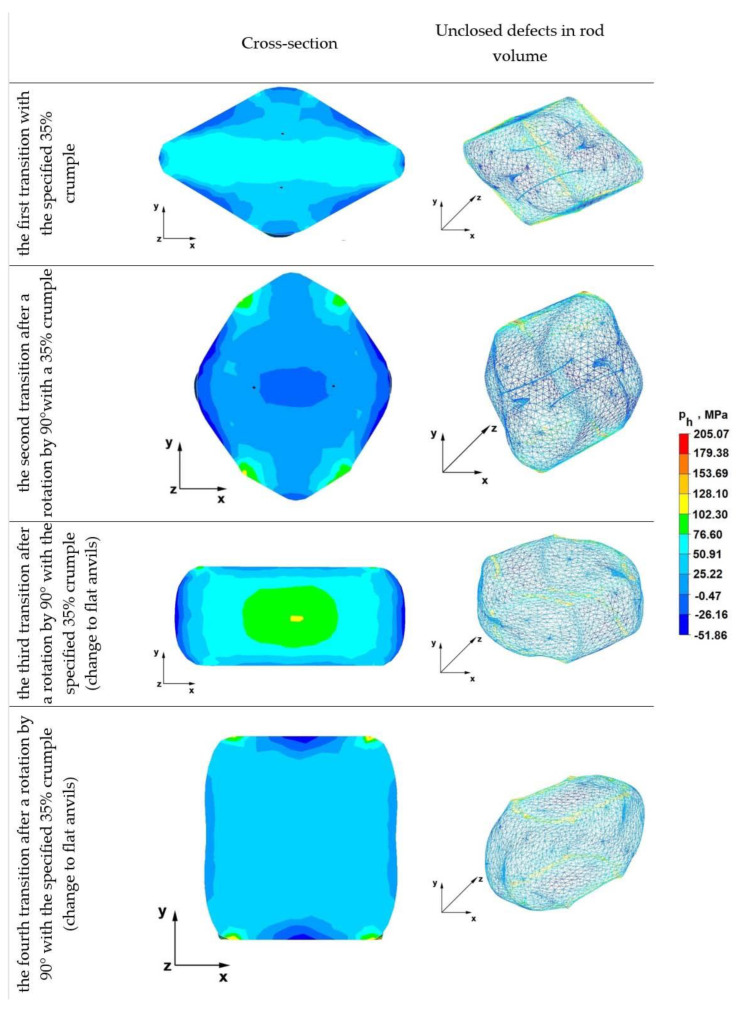
Hydrostatic pressure distribution in rhombic anvils.

Figure 11 shows the distribution of hydrostatic pressure values obtained during numerical simulations of the operation of elongating the rod from the Zr alloy in rhombic anvils.

Analyzing the resulting data, it can be stated that after the first forging transition, an axial discontinuity with an original diameter of 10 mm was completely welded, which was not achieved during the implementation of the first crumb in the assembly of rhombic flat and flat anvils. It can be stated that in this case, the axial defect was welded, because the direction and return of the pressure forces coming from the working side surfaces of the upper and lower rhombic anvil were directed towards the axis of the forged rod, i.e., the pressure force was intensified in this area. The hydrostatic pressure along the x-axis of the deformed rod was 51 MPa, while in the apex areas of diamond anvils, it was 25 MPa. Too small a value of hydrostatic pressure mixing in the range of 7–22 MPa does not provide conditions for welding discontinuities located in the described areas.

The data in the second transition show that despite the application of another crumple, after turning the rod by 90°, the existing two discontinuities remaining after the first forging transition were not completely welded. In the area of discontinuities, there was no hydrostatic pressure, and the average stress values ranged from 0.47 to 25 MPa, i.e., there were tensile stresses hindering the discontinuity welding process.

In the third pass, as can be seen from the figure, there were no unwelded discontinuities, and the pressure values in the middle area of the road were in the range of 77–102 MPa. Outside the axial zone, these values ranged from 25 to 51 MPa. This distribution of hydrostatic pressure has a very beneficial effect on the discontinuity welding process. The use of more than one anvil composition in the zirconium alloy rod elongation operation favorably affects the discontinuity welding process, because the use of two different anvil compositions with different working surfaces allows for the introduction of a different nature of metal flow (change in directions and reversals of friction forces and pressure forces) during one elongation operation. It is worth mentioning that during the execution of a single elongation operation using only one anvil assembly, it is not possible to obtain differentiated metal flow kinematics in the entire volume of the deformed rod.

From the analysis of the fourth transitions, it follows that in the majority of the cross-sectional area of the forged rod, a favorable and even hydrostatic pressure of 25 MPa was obtained.

## 7. Analysis of the Distribution of Deformation Intensity Values during Elongation Operations

In each of the forging elongation operations of ingots after the casting process of metallic alloys, carried out in order to weld metallurgical discontinuities such as middle porosity and metallurgical voids, in the area of the discontinuities, especially in axial areas, it should be sought to obtain the highest value of deformation intensity due to the relatively large areas of the surfaces of these discontinuities. However, in production practice, when carrying out the rod-lengthening operation, it is difficult to introduce a deformation state characterized by high deformation values, especially compressive strains in the axial zones of the forging, as well as in other local zones of the deformed bar, where, as a rule, there are also small discontinuities. In order to achieve the intended character of deformations, favoring the welding of discontinuities, the authors proposed that forging elongation operations should be carried out in rhombic anvils, thanks to which, it is possible to control the direction, return and value of the friction force vectors and pressure forces acting in the deformation zone and to select the appropriate values of the main technological parameters of the elongation operation.

### 7.1. Analysis of the Distribution of the Deformation Intensity Values during the Elongation Operation in Rhombic Flat Anvils

**Figure 12:**
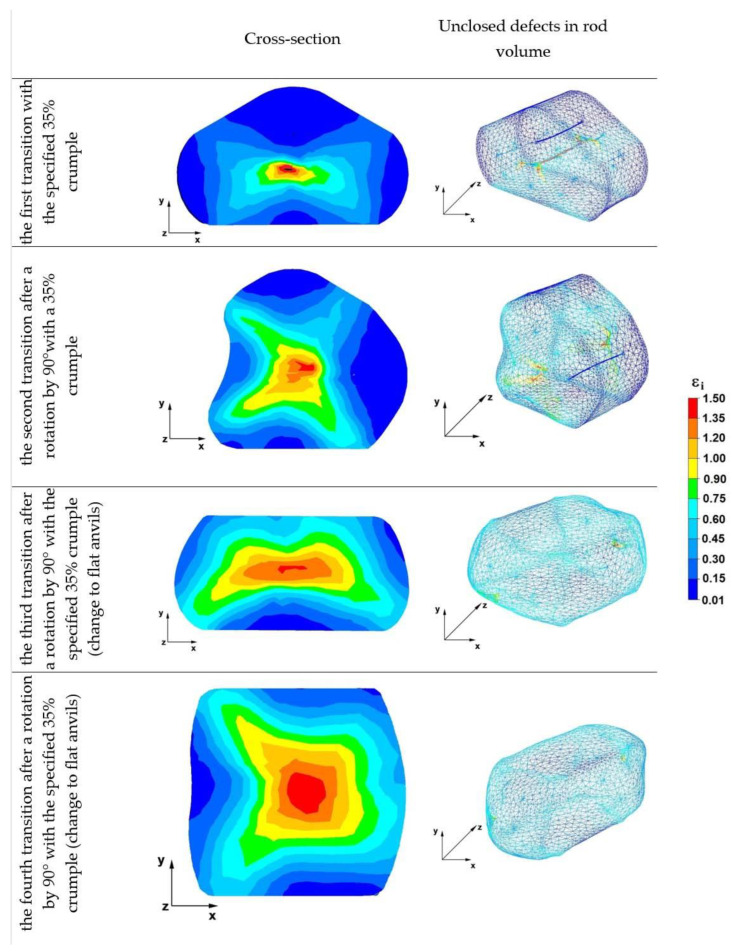
Effective strain distribution in rhombic flat anvils.

Figure 12 shows the deformation intensity distributions obtained during numerical simulations of the Zr alloy rod-lengthening operation in rhombic flat anvils.

In the first forging transition in the axial zone of the deformed rod, the deformation intensity values ranged from 1.00 to 1.50 and did not lead to the complete welding of the axial discontinuity by the angle of 90° and reimplementation of the 35% crumple; the deformation intensity values were the same, and the axial discontinuity was completely welded, which is visible in the second transition. On the other hand, in the case of discontinuities located at a distance of 25 mm from the axis of the deformed rod, after the first forging transition, two discontinuities remained under the direct action of the upper rhombic anvil (first transition). After the second forging transition, one discontinuity remained along the x-axis direction on the right side of the deformed rod. It happened because both in the first forging transition and the second, in these areas, there was a deformation intensity with small values ranging from 0.01 to 0.15. Little or negligible intensification of deformations in the area of discontinuities during forging operations does not contribute to the welding of discontinuities.

The analysis of the data of the third transition shows that after the third forging transition with 35% crumple, after the rod was heated to 950 °C and rotated by 90°, all discontinuities were welded. This was the result of a higher value of the deformation intensity in the areas where there were previously unwelded discontinuities. The values of the deformation intensity ranged from 0.45 to 0.57 and differed from the values occurring after two forging transitions.

Based on the data of fourth transition, it can be concluded that after the fourth and last forging transition, there was still a favorable distribution of deformation intensity favoring the welding of discontinuities; in the central area, the value of the deformation intensity ranged from 0.90 to 1.50, while in areas distant from the rod axis, it was within the limits of 0.45–0.90.

### 7.2. Analysis of the Distribution of the Deformation Intensity Values during the Elongation Operation in Flat Anvils

**Figure 13:**
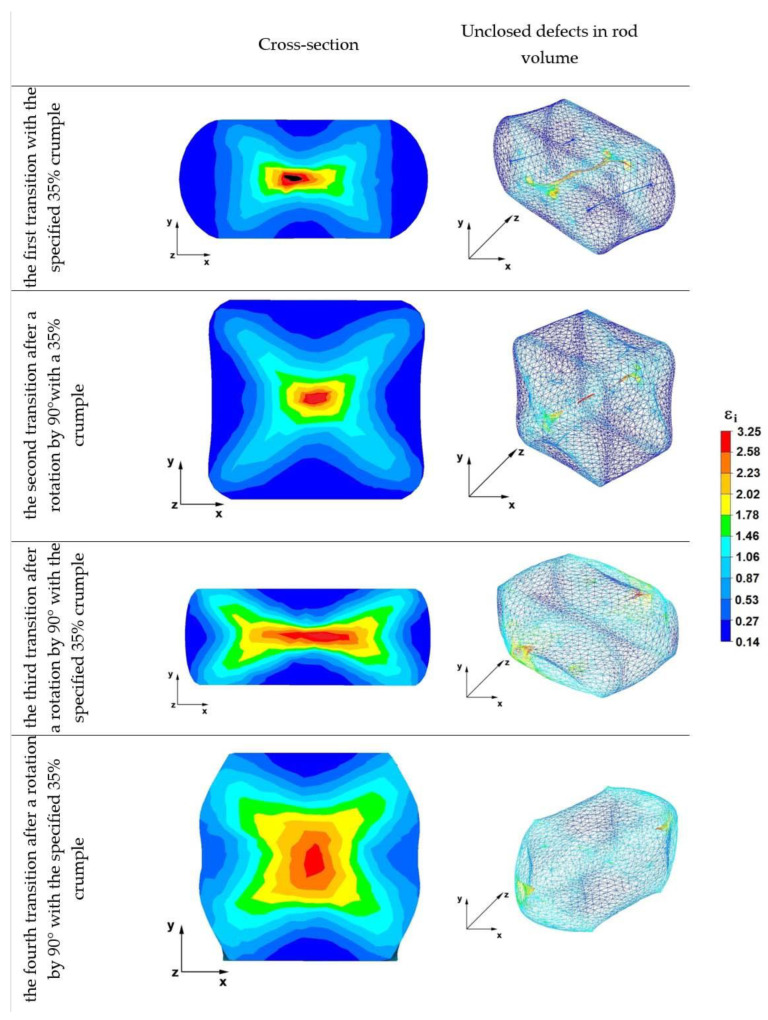
Effective strain distribution in flat anvils.

Figure 13 shows the deformation intensity distributions obtained during numerical simulations of the elongation operation of the Zr alloy rod in flat anvils.

The data of first transition show that in the axial zone of the deformed rod, the deformation intensity value was 3.25, but this did not result in welding the discontinuity in the rod axis. The two discontinuities originally 25 mm away from the axis of the forged rod on both sides along the x-axis were also not completely welded, because the value of the deformation intensity was 0.14. This was due to the free movement of the material towards the surface that was not limited by tools, where tensile deformations were mostly present. Only the discontinuities under the direct action of the surface of flat anvils, where compressive deformations prevailed, were completely welded.

By analyzing the data of the second transition, it can be concluded that in the axial zone of the deformed rod after the second forging transition (the rod was turned by an angle of 90° and the value of the crumple was 35%), a high intensity of deformation was maintained, which ultimately resulted in welding the axial discontinuity; the value of the deformation intensity in this area was 3.25. However, also after the second forging transition, in the areas of the deformed rod under the influence of the working surfaces of the flat anvils, the intensity of the deformation was relatively low, which resulted in the remaining discontinuities not being completely welded. The value of the deformation intensity in this area was still equal to 0.14.

On the basis of the analysis of the distribution of the deformation intensity values presented in figures of the third transition, it can be seen that all discontinuities were welded.

The values of the deformation intensity in the central area of the deformed rod were within 2–3, while in the area distant from the rod axis, they ranged in the range of 0.87–1.06. The exceptions were the areas located right next to the contact surface of the material with the anvil and the areas not limited by the working surfaces of the anvils along the x-axis. The value of the deformation intensity was small there, amounting to 0.14.

The data in the figures of the fourth transition show that the distribution of the deformation intensity value after the fourth transition did not change compared to the state occurring after the third forging transition. In the central part of the rod, the values of the deformation intensity were recorded in the range 1.46–3.25, while in the area distant from the rod axis, the values were within the range of 0.87–1.06.

### 7.3. Analysis of the Distribution of the Deformation Intensity Values during the Elongation Operation in Rhombic Anvils

**Figure 14:**
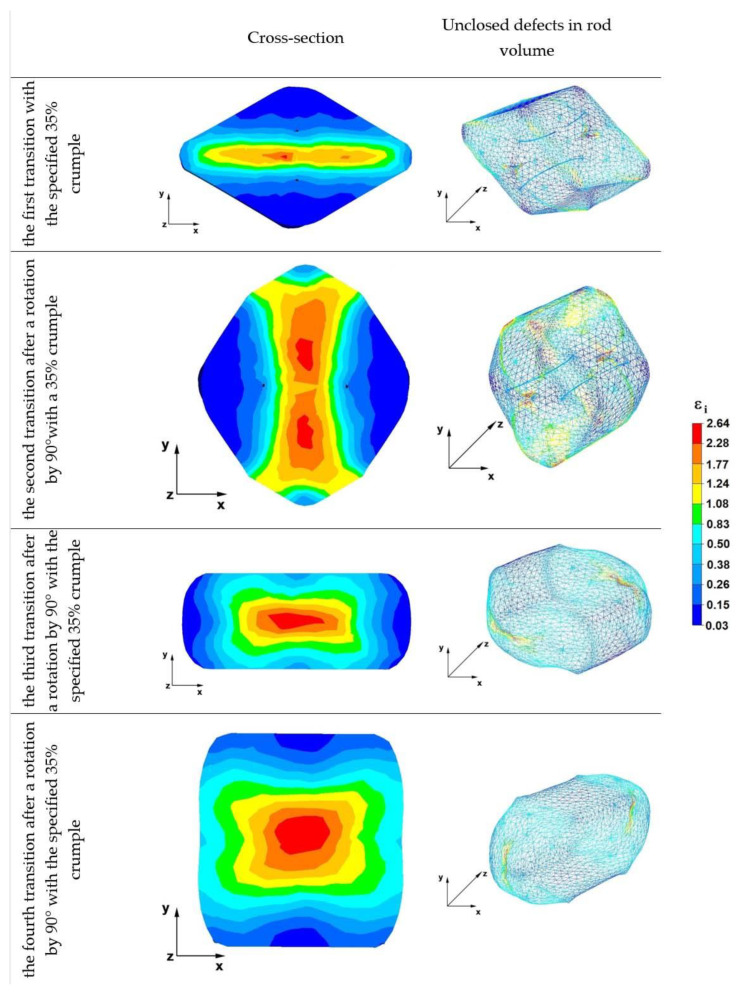
Effective strain distribution rod in rhombic anvils.

Figure 14 shows the deformation intensity distributions obtained during numerical simulations of the operation of elongating the Zr alloy rod in rhombic anvils.

Analyzing the data first transition, it can be concluded that the largest axial discontinuity was welded after the first forging transition carried out in rhombic anvils. The value of the deformation intensity in this area ranged from 1.77 to 2.28, while the discontinuities located under the tops of the anvil working surfaces remained unwelded. The values of the deformation intensity in these areas ranged between 0.15 and 0.36. It follows that the intensification of deformations was too small to lead to welding of the discontinuities in the deformed zirconium alloy rod. The use of rhombic anvils, due to the shape of their working surfaces, affected the favorable distribution of the intensity of deformations in the axial areas of the deformed rod. In the areas under the anvil tips, the value of the deformation intensity was insufficient for the welding of metallurgical discontinuities. The pressure exerted on the forging by the rhombic anvils was almost entirely exerted in the direction of the axis of the forged rod.

The data of the second transition show that in the axial zone of the deformed rod, after rotation by an angle of 90°, the deformation intensity distribution favorable for the process of welding the discontinuities was maintained. The values of the deformation intensity ranged between 1.24 and 2.28. After both the first and the second forging transition, the remaining discontinuities were not welded due to the unfavorable distribution of the deformation intensity, the values of which were small and ranged from 0.15 to 0.36. The material flowed freely along the x-axis into spaces not limited by the working surfaces of rhombic anvils. Tensile deformations occurred in these areas, which adversely affected the process of welding the discontinuities.

In the third transition, after replacing rhombic anvils with flat anvils and heating the rod to the initial forging temperature and rotating it by 90°, the remaining discontinuities were welded. In the central area of the deformed rod, the deformation intensity values ranged from 1.08 to 2.64, while in the remaining areas, the deformation intensity values ranged from 0.38 to 1.08.

After the fourth and last forging transition, the distribution of the deformation intensity values in the metal was very similar to that in the third transition. The deformation intensity values, especially in the central part of the deformed rod, were very favorable and ranged from 1.08 to 2.64, while in other areas, except for small areas at the contact of the deformed material with the tool, the deformation intensity values fluctuated within 0.38–1.08.

## 8. Influence of the Shape of the Anvils on Changes in the Volume of Non-Continuity, Hydrostatic Pressure and the Stage-Forming Intensity in the Rate of the First Two Forging Transitions

**Figure 15:**
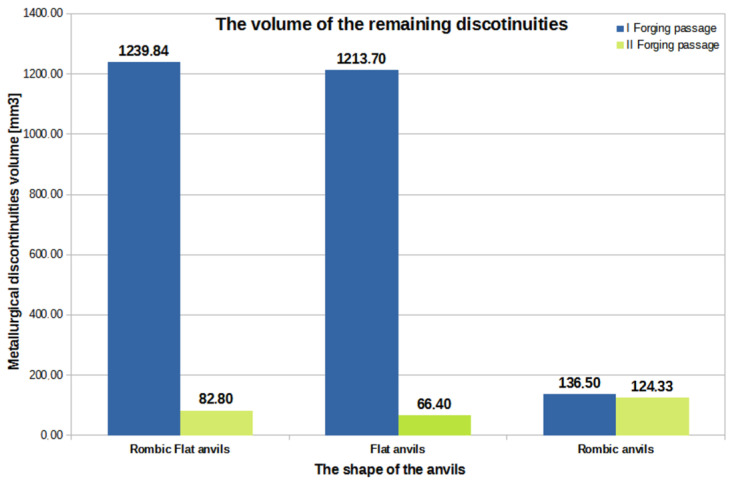
Graph of the total volume of unwelded discontinuities in the volume of a forged zirconium alloy rod for two forging transitions in relation to individual anvil compositions.

**Figure 16:**
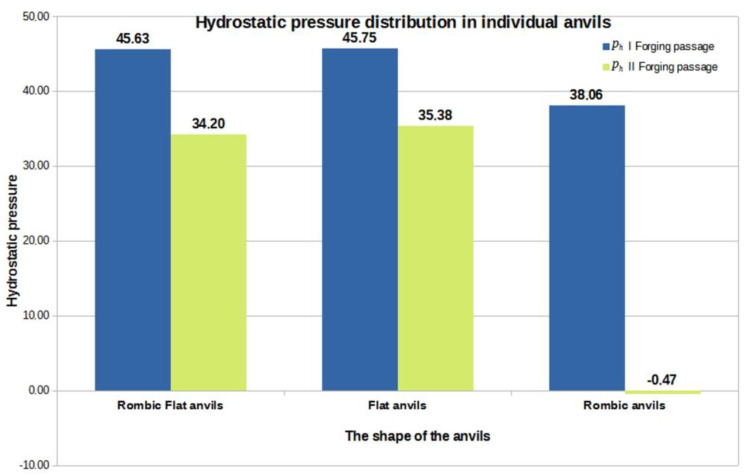
The graph of the arithmetic mean value of the hydrostatic pressure around the unwelded discontinuities in relation to the individual anvil compositions.

**Figure 17:**
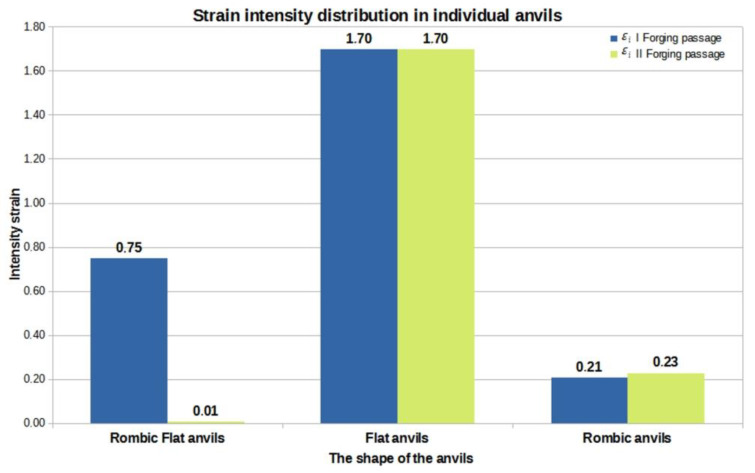
A graph of the arithmetic mean value of the deformation intensity occurring around unwelded discontinuities in relation to individual anvil compositions.

Figure 15, Figure 16 and Figure 17 show graphs of the total volume of non-welded discontinuities, arithmetic mean values of hydrostatic pressure and arithmetic mean values of deformation intensity occurring around non-welded discontinuities for all the anvil compositions analyzed in the work, divided into the first and second forging transitions.

By analyzing the data in Figure 15, Figure 16 and Figure 17, the authors try to explain why in individual anvil compositions such total values of the volume of unwelded discontinuities were obtained. It is known that the best results in terms of welding discontinuities in the deformed metal can be obtained when the temperature in the areas of the metal around the discontinuities is sufficiently high to initiate the welding process and when high values of compressive stresses and high deformation intensity values are also present in the welded discontinuities.

The data in Figure 15 show that after the first forging transition for the rhombic flat anvils, the reduction in the total volume of unwelded discontinuities was 1587.60 mm^3^, in relation to the initial value of 4239 mm^3^.

By analyzing the data in Figure 15, it can be concluded that the best results in terms of welding the metallurgical discontinuities during the operation of elongating zirconium alloy rods after the first and second forging transitions were obtained for rhombic flat and flat anvils. Although in the first forging transition, for these compositions, large values of the total volume of unwelded discontinuities ranging from 1213.70 to 1239.84 mm^3^ were obtained, already in the second forging transition, after turning the deformed bar by 90° and resetting the 35% relative crumple, the value of the total volume of unwelded discontinuities significantly decreased and ranged from 66.40 to 82.80 mm^3^. The research shows that rhombic anvils can also be used to weld metallurgical discontinuities. Here, in contrast to the rhombic flat and flat anvils, after the first forging transition, a reduction in the total volume of discontinuities to the value of 136.50 mm^3^ was noted. However, after the second forging transition, the area of unwelded discontinuities decreased to the value of 124.33 mm^3^. The total, the initial value of the volume of all modeled discontinuities was equal to 4239 mm^3^.

The data in Figure 16 show that in the first forging transition for the rhombic flat and flat anvils, the arithmetic mean value of the hydrostatic pressure in the area around the unwelded discontinuities was equal to 46 MPa. It can be assumed that in the second forging transition, while maintaining this average pressure value, all discontinuities would weld, or their total volume would be reduced. The data presented in Figure 16 show that the arithmetic mean value of the hydrostatic pressure did not increase but decreased to the value of 35 MPa, which, however, did not inhibit the process of welding the discontinuities. Based on the analysis of the elongation operation in rhombic anvils, it was found that in the second forging transition, there was a significant decrease in the value of hydrostatic pressure, which did not significantly affect the welding process of metallurgical discontinuities.

The analysis of the data in Figure 17 shows that the most favorable arithmetic mean value of the deformation intensity after two forging transitions was obtained for folding flat anvils, and it was equal to 1.70. This contributed to the reduction in the total volume of discontinuities after the second forging transition from 4239 mm^3^ to 66.40 mm^3^. The arithmetic mean values of the deformation intensity calculated for the rod elongation operation in the remaining anvil compositions had much lower values.

Summing up, on the basis of the results shown in Figure 15, Figure 16 and Figure 17, it can be concluded that the higher the hydrostatic pressure values that occurred in the areas around the metallurgical continuity, the more effective the welding process of these discontinuities was. Additionally, small drops in the value of hydrostatic pressure did not inhibit the process of welding the discontinuities. Considering the zirconium alloy elongation operations in three different anvil compositions analyzed in this paper, it can be concluded that it is favorable, in terms of welding the discontinuities, to maintain the deformation intensity value at the level of 1.70 and higher in the area of welded discontinuities.

## 9. Final Conclusions

Based on the analysis of the results of the conducted research, the following conclusions were drawn:

1. The use of figured (cut-out) anvils in the hot forging process significantly affects the welding of internal metallurgical discontinuities in local zones of deformed zirconium alloy forging, depending on the geometry of the anvils used.
2. To weld metallurgical discontinuities, it is necessary to carry out forging with the maximum possible values of relative reduction up to 40%.
3. The implementation of forging with significant reductions contributes to the formation of favorable compressive deformations in the local areas of the forging, which create conditions for welding metallurgical discontinuities.
4. When forging a zirconium alloy ingot using flat and rhomboid flat anvils, high values of hydrostatic pressure are noted, which contributes to a significant reduction in the total volume of metallurgical defects.
5. The axial discontinuity of the largest size was welded using rhombic anvils in the first forging pass.
6. When using all variants of anvils, the welding of metallurgical discontinuities was noted in the third forging pass.
7. As a result of the research, it was found that the complete welding of metallurgical discontinuities is achieved by using figured (cut-out) anvils in the first passes of forging and in subsequent passes of forging flat anvils.
8. The authors intend to carry out the verification of the obtained modeling results under laboratory conditions in the near future.

## Figures and Tables

**Table 1:** Edge conditions for determining the flow stress σ_p_ of the M5 alloy on the Gleeble 3800 plastometer under hot forging conditions.

N	έ, s^−1^	ε	T °C		
1	0.5	≤0.9	950	850	770
2					
3					
4	5				
5					
6					

**Table 2:** Hensel–Spittel equation data.

Alloy	Values of the Coefficients of the Hensel–Spittel Equation									Average Approximation Error
	A	m1	m2	m3	m4	m5	m7	m8	m9	
M5	0.0308	−0.0125	0.2969	−0.3725	1.7340	0.0038	−2.5447	0.0007	2.7487	10%

## Data Availability

Not applicable.
